# Functionalized nanomedicines for co-targeting of multiple pathways to efficiently treat thyroid cancer: prospects and challenges

**DOI:** 10.3389/fphar.2026.1796656

**Published:** 2026-05-15

**Authors:** Haidi Chu, Dong Wang

**Affiliations:** 1 Department of Thyroid Surgery, Yantaishan Hospital, Yantai, Shandong, China; 2 Department of Head and Neck Surgery, Affiliated Hospital of Qingdao University, Yantai Yuhuangding Hospital, Yantai, Shandong, China

**Keywords:** combination therapy, co-targeting, functionalized nanomedicines, multi-pathway, therapeutic resistance, thyroid cancer

## Abstract

Complex molecular signaling networks regulate the development of thyroid carcinoma by providing neoplastic cells with the ability to adapt, survive, and multiply. Conventional single-target systems of therapeutic administration are often insufficient to provide adequate clinical efficacy, due to redundancy in signaling pathways, and compensatory response and resultant systemic toxicity. Functionalized nanomedicines are a novel modality that can potentially overcome these challenges by allowing simultaneous modulation of multiple cascades of oncogenic pathways. This review looks into the molecular pathways that are involved in the development and progression of thyroid cancer; special attention is paid to the central signaling strings which regulate cell proliferation, cell survival, angiogenesis and apoptosis. The focus is made on the co-targeting of these pathways by the means of fine tumor specificity and controlled drug delivery. This review outlines modern developments in the field of functionalized nanomedicines that deal with thyroid carcinoma, with a particular focus on the potential and the challenges of how to target two or more molecular conduits simultaneously. As compared to the previous reviews of subjects that examine single targets or single treatments in the management of thyroid cancer, our discussion predestines the novel approach of using functionalized nanomedicines to concomitantly probe into multiple signaling pathways-an approach that is extremely important in addressing the challenges of therapeutic resistance and improving clinical efficacy. The future directions are also outlined, and the prospects of personalized and intelligent nanomedicine-based solutions to change the landscape of therapeutic options of thyroid carcinoma can be viewed.

## 1 Introduction

A steady increase in the incidence of thyroid cancer can be observed globally in the past decades, which makes this type of cancer one of the most commonly diagnosed malignancies in the world. The study of the GLOBOCAN database data demonstrates that over half a million new cases of thyroid cancer were reported all over the world in 2020, which makes the disease one of the top ten prevalent cancers (Cao et al., 2024; Lyu et al., 2024). Significantly high percentages of these cases were discovered in the country where the level of Human Development Index is high and very high, and they collectively represent more than ninety percent of new diagnoses (Khazaei et al., 2020). The Western Pacific region exhibited the greatest disease burden with almost half of the reported disease burden though it constitutes a quarter of the world population. It is also projected that by 2040, the incidence and mortality will increase significantly with projections becoming very steep in low-HDI areas. It is worth noting that it is in the African region that the relative growth in cases and deaths caused by disease is likely to increase the most. Incidence rates are the highest in socioeconomically advanced areas, whereas the mortality is disproportionately high in the countries with low HDI, which may be due to the inequality in healthcare access, early diagnosis, and availability of treatments, but also to the effects of environmental and nutritional risk factors such as exposure to ionizing radiations and lack of iodine. These observations highlight the necessity of globally balanced interventions that will help bring more thyroid cancer to the early phase of disease awareness, access to treatment, and general prognosis of the disease regardless of geographical and socioeconomic backgrounds (Lyu et al., 2024; Ji et al., 2025).

The simultaneous dysregulation of a combination of metabolic and signaling pathways that interactively shape tumor growth and immune evasion controls cancer development and resistance to treatment. Although immunotherapeutic approaches have significantly expanded the list of treatment modalities, adaptive metabolic reprogramming in the tumor cells and immune cells inhabiting the tumor microenvironment (TME) often diminishes the effectiveness of immunotherapeutic approaches in the long-term (You et al., 2023). The major pathways such as glucose metabolism, amino-acid utilization, lipid metabolism, nucleotide biosynthesis, and mitochondrial activity are all concomitantly disturbed to favor the survival of tumors whilst inhibiting antitumor immune responses. The interacting network generates an immunosuppressive metabolic environment that involves nutrient competition, hypoxia and acidic stress which interact to suppress the activity of cytotoxic immune cells and induce regulatory immune phenotypes (Augustin et al., 2020). Metabolites produced by the tumors add to pathway crosstalk that enables immune evasion and therapy resistance. Due to their redundancy and interdependence, single-target therapies are often insufficient. Modulation of several pathways, through providing therapeutic approaches that allow its coordination has thus become a matter of growing interest. Nanomedicines provide flexibility in that they enable tumor specific co-delivery of metabolic inhibitors and immunomodulatory agents that can be used to target multiple pathways in order to overcome resistance and increase therapeutic efficacy (Zhao et al., 2025).

**FIGURE 1:**
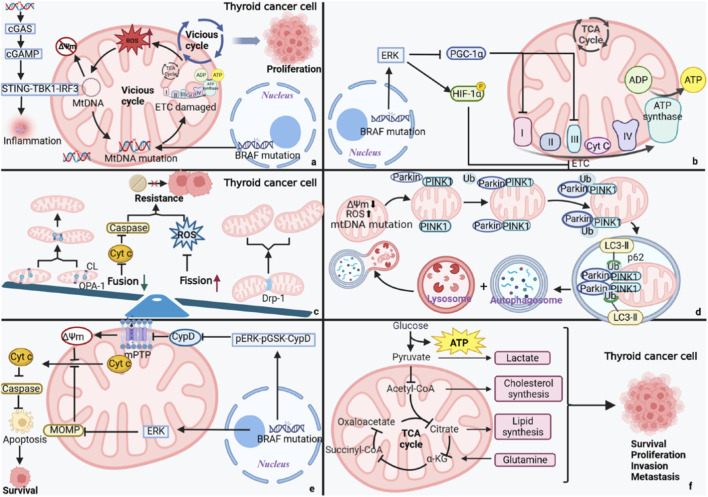
Schematic illustration of mitochondrial dysfunction–driven mechanisms contributing to thyroid cancer (TC) pathogenesis. **(a)** Mitochondrial DNA mutations cause a disruption of the electron transport chain (ETC.) leading to an unwanted overproduction of reactive oxygen species (ROS) creating a self-reinforcing cycle in support of TC cell proliferation. **(b)** Cellular energy production is impaired by structural and functional defect of the, ETC. **(c)** Impaired dynamics of mitochondria fusion, as well as increased fission, facilitates resistance to therapy in TC cells. **(d)** The changes in autophagic processes within the mitochondria also contribute to the breakdown of the mitochondrial homeostasis. **(e)** Reduced mitochondrial membrane permeability inhibits apoptotic signaling, which allows TC cell survival. **(f)** Metabolic reprogramming enhances invasion, metastasis and proliferative endurance of TC cells. Red arrows indicate the upregulated pathways, molecular functions or metabolic activities, and green arrows illustrate the downregulated processes. (Zhang Y. et al., 2025).

The development and resistance to treatment of thyroid cancer is facilitated by the dysregulation of several oncogenic signaling and metabolic pathways as a concerted failure, and not by the change in a molecular axis. The schematic description of the mechanisms of pathogenesis of thyroid cancer that are the result of mitochondrial dysfunction is shown in Figure 1. Disruption of the electron transport chain (ETC.) by mutations of mitochondrial DNA causes the overproduction of reactive oxygen species, a situation that creates a self-sustaining loop and supports the proliferation of tumor cells. The functional and structural damage on the, ETC., leads to the impaired cellular energy production. Functional pathways, including MAPK/ERK and PI3K/Akt/mTOR, are important in the regulation of tumor cell growth, survival and differentiation, and they simultaneously contribute to metabolic pathways maintaining the growth of tumors. Besides these signaling cascades, there is extensive metabolic reorganization of thyroid cancer cells, characterized by increased glycolysis, changes in amino-acid metabolism, lipid metabolism, and mitochondrial dysfunction, which combine to promote tumor aggressiveness and immune suppression in the TME (Li et al., 2022). The interaction between these pathways produces compensatory signaling pathways and metabolic plasticity, allowing tumor cells to avoid the force of therapy and decreasing the power of monotherapies (Ahuja and Zaheer, 2025). Moreover, antitumor immune responses are also impaired by the metabolic competition and tumor-derived metabolites in the TME, strengthening resistance mechanisms (Li S. et al., 2025). Since signaling and metabolic pathways are broadly crosstalked in thyroid cancer, treatment paradigms that have the ability to co-target multiple pathways are now viewed as a necessity. Functionalized nanomedicine should offer a viable solution since it can be used to deliver pathway-specific inhibitors and metabolic modulators with greater tumor selectivity, providing a rational approach to resistance by-pass and improve therapeutic outcomes (Alshahrani et al., 2025).

This systematic review targets functionalized nanomedicines when used in treatment of thyroid cancer with an emphasis on those methods which can co-target several oncogenic and metabolic pathways. Although some of the published reviews discuss the therapeutic targets under single-pathway interventions when treating the thyroid cancer, the review focuses on the emerging approach of the advanced functionalized nanomedicines which can target more than one signaling pathway to treat thyroid cancer. This novel multi-target approach is of vital importance for overcoming the complexities involved in the resistance mechanisms in thyroid cancer to enhance the treatment efficacy. By integrating current molecular insights and emerging nanotherapeutic strategies, this aims to highlight approaches that can enhance treatment specificity and overcome therapeutic resistance.

## 2 Molecular pathways involved in thyroid cancer progression

The progression of the thyroid cancer is mainly driven by an intricate network of the complex molecular pathways, which regulate the proliferation, survival, differentiation, and metastasis of the cells. The most prominent signaling cascades that are implicated in the tumorigenesis of the thyroid cancer the MAPK/ERK, PI3K/Akt/mTOR and the angiogenic pathways (Li et al., 2022; Lee et al., 2020; Ersahin et al., 2015; Song et al., 2020). Also, the BRAF mutations, and the apoptosis and cell cycle dysregulation are also involved in the progression of the thyroid cancer (Trovisco et al., 2005; Zaman et al., 2019). These factors are discussed in detail in the following.

### 2.1 MAPK/ERK pathway

**FIGURE 2:**
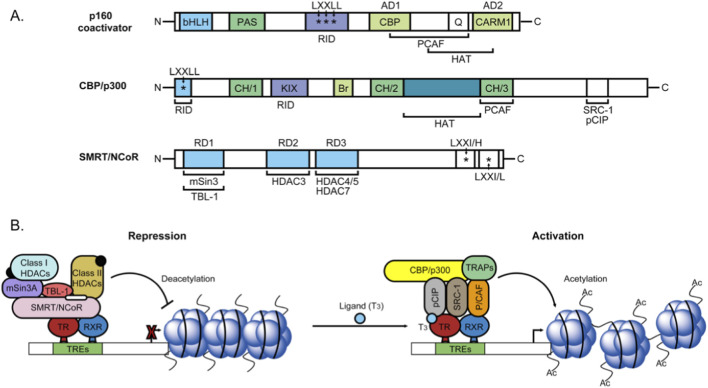
Overview of structural organization of thyroid hormone receptor (TR) coactivators and corepressors, along with their roles in the regulation of gene transcription **(A)** Biochemical studies have demonstrated that TRs interact with multiple coactivator complexes, most prominently members of the p160 protein family, as well as histone acetyltransferases (HATs) such as CREB-binding protein (CBP) and p300. In contrast, in the absence of ligand, TRs exhibit strong association with corepressor complexes, including nuclear receptor corepressor (NCoR) and silencing mediator for retinoic acid and thyroid hormone receptors (SMRT) **(B)** Regulation of gene expression by TRs occurs in a ligand-dependent fashion. TRs form heterodimers with retinoid X receptors (RXRs) and bind to thyroid hormone response elements (TREs) within target gene promoters. The ligand-binding domain (LBD) facilitates interaction with triiodothyronine (T3), while the DNA-binding domain (DBD) confers high specificity for TRE sequences. Through direct or indirect interactions with transcription factors, coactivators, transcriptional intermediary factors (TIFs), and corepressors, TRs modulate downstream gene transcription. Adapted from the reference (Liu et al., 2019).

Thyroid hormones, thyroxine (T4) and triiodothyronine (T3), are key regulators of thyroid cancer progression through non-genomic signaling, acting primarily *via* cell membrane-bound integrin αvβ3 receptor. The structural organization of coactivators and corepressors for thyroid hormone receptor and their role in the regulation of gene transcription is illustrated in Figure 2. Biochemical studies have revealed that the thyroid hormone receptors interact with multiple coactivator complexes, most prominently members of the p160 protein family, as well as histone acetyltransferases (HATs) such as CREB-binding protein (CBP) and p300. In contrast, in the absence of ligand, TRs exhibit strong association with corepressor complexes, including nuclear receptor corepressor (NCoR) and silencing mediator for retinoic acid and thyroid hormone receptors (SMRT). The interaction of T3 and T4 bypasses classical nuclear thyroid hormone receptors (TRs) and initiates downstream activation of multiple oncogenic pathways, including mitogen-activated protein kinase/extracellular signal-regulated kinase (MAPK/ERK) and phosphoinositide 3-kinase/protein kinase B (PI3K/Akt), thereby linking systemic thyroid hormone activity to cellular proliferation, survival, and tumor microenvironment modulation. The binding of the thyroxine (T4) and the triiodothyronine (T3) to the unique integrin αvβ3 domains activates dual the oncogenic signaling axes including mitogen-activated protein kinase/extracellular signal-regulated kinase (MAPK/ERK) and phosphoinositide 3-kinase/protein kinase B (PI3K/Akt), orchestrating tumor proliferation, cell cycle progression, and survival (Liu et al., 2019). The MAPK/ERK pathway promotes transcription of early-response genes, including FBJ murine osteosarcoma viral oncogene homolog (FOS), Jun proto-oncogene (JUN), and MYC proto-oncogene, bHLH transcription factor (MYC), and upregulates Cyclin D1, facilitating the G1-to-S phase transition, while PI3K/Akt signaling enhances survival through downstream effectors such as mammalian target of rapamycin (mTOR), forkhead box O (FOXO), phosphatase and tensin homolog (PTEN), and glycogen synthase kinase-3 beta (GSK-3β) (Ersahin et al., 2015). Collectively, these pathways induce angiogenesis *via* hypoxia-inducible factor-1 alpha (HIF-1α) stabilization and vascular endothelial growth factor (VEGF) upregulation, promote metastasis and invasion through matrix metalloproteinases (MMP-2/9) and Rho GTPase–mediated cytoskeletal remodeling, inhibit apoptosis by phosphorylating Bcl-2-associated death promoter (BAD) and activating B-cell lymphoma 2 (Bcl-2), and facilitate immune evasion *via* upregulation of programmed death-ligand 1 (PD-L1) (Jing et al., 2019). Therapeutically, T4 antagonist tetraiodothyroacetic acid (Tetrac) and its nanoformulation, nano-diamino-tetrac (NDAT), competitively inhibit αvβ3 binding, suppress MAPK/ERK signaling, reduce tumor proliferation, angiogenesis, and MMP activity, and demonstrate promising potential as targeted anti-metastatic agents within tumor microenvironment (Yalcin et al., 2010).

### 2.2 PI3K/Akt/mTOR pathway

The phosphoinositide 3-kinase/protein kinase B/mammalian target of rapamycin (PI3K/AKT/mTOR) signaling axis is one of the most frequently dysregulated pathways in cancer, playing a central role in tumor survival, proliferation, metastasis, metabolism, angiogenesis, epithelial–mesenchymal transition (EMT), apoptosis, autophagy, ferroptosis, glycolysis, immune evasion, and therapeutic resistance (He et al., 2021). Aberrant activation or mutation of such major components as PIK3CA, PTEN (phosphatase and tensin homolog), mTOR promote carcinogenesis, suppress apoptosis and ferroptosis, increase EMT and angiogenesis, and develop resistance to chemotherapy, radiotherapy, and immunotherapy (Rascio et al., 2021). Noncoding RNAs also interact with this axis, which are epigenetically regulated to maintain their activity, which provides possible targets of RNA-based diagnostics and therapies. Small molecules, phytochemicals, and nanoparticle-based systems of PI3K/AKT/mTOR can be used therapeutically to prevent tumor progression and overcome resistance (Beigi et al., 2025). More specific and possibly able to avoid therapy resistance, advanced methods, such as PROTACs (Proteolysis Targeting Chimeras), which degrade important proteins, and metamorphic, which disrupt noncatalytic protein functions, are more specific (Li and Song, 2020). PI3K/AKT/mTOR inhibition combined with immunotherapy, including programmed death-1/programmed death-ligand 1 (PD-1/PD-L1) checkpoint blockade, is able to reprogram the tumor microenvironment, promote immune-mediated tumor elimination, and work better (Ladoire et al., 2023). Future studies are directed at an explanation of cross talk between this axis and other oncogenic or immune-regulatory pathways, clarification of the dual roles of the autophagy and apoptosis that are dependent on circumstances and biomarker-based precision methods to optimize clinical translation.

### 2.3 Angiogenic pathways

The same happens with tumor-associated macrophages (TAMs) with thyroid endocrine tumors. TSH-suppression is one of the essential measures after surgery to avoid the recurrence of the tumor following the differentiated thyroid carcinoma (DTC). TSH can be found to promote the release of vascular endothelial growth factor A (VEGF-A) through phosphoinositide 3-kinase/protein kinase B/mammalian target of rapamycin (PI3K/AKT/mTOR) or extracellular signal-regulated kinase (ERK) signifiers (Zhang et al., 2023). This, subsequently, increases the expression of macrophage markers, CD31, CD163 and chemokine CXCL8, which encourages macrophage infiltration, tumor angiogenesis and proliferation (Owen et al., 2013). In the clinical setting, increased VEGF-A levels correlate with the increased incidence of remote metastasis and decreased disease-specific survival, which reflects the importance of TAM-mediated signaling in the development of thyroid cancer (Cotechini et al., 2021).

### 2.4 BRAF mutations

BRAF (B-Raf proto-oncogene, serine/threonine kinase) is used to encode a serine-threonine protein that passes signals to the nucleus through the cell membrane, which stimulates cell growth and proliferation (Zaman et al., 2019). BRAF, which was initially discovered in 1982, is also an essential mediator of thyroid cancer development, as it is enough to trigger the growth and evolution of tumors (Xing, 2013). Activation results in progressive degradation of the variousiation markers, such as thyroglobulin (Tg) and sodium-iodide symporter (NIS) which inhibit iodine uptake and is the cause of radioiodine-refractory disease (Spit et al., 2014). The most frequently occurring mutation is BRAFV600E that entails the replacement of valine (V) with glutamic acid (E) at codon 600 in exon 15. The mutation has a prevalence of about 99 percent of all thyroid cancers and of 58.6 percent of differentiated thyroid cancers (DTC), and 45 per cent of anaplastic thyroid carcinomas (ATC) (Trovisco et al., 2005).

### 2.5 Apoptosis and cell cycle dysregulation

Mutations in the ATM (ataxia-telangiectasia mutated serine/threonine kinase) and in the sense of the DNA mismatch repair (MMR) pathway, such as MutL homolog 1 (MLH1), MutS homolog 2 (MSH2) and MutS homolog 6 (MSH6), have been primarily truncated in anaplastic thyroid carcinomas (ATCs) (Santos et al., 2018). DNA repair dysfunction is likely to allow accumulation of mutations and chromosomes defects leading to genomic instability, which is also observed in other malignancies (Wei et al., 2016). Truncating MMR mutations were found in cells: The functional thyroid cancer cell lines FTC133 and T243 have shown increased tumor mutation burdens with T243 showing microsatellite instability by short tandem repeat (STR) profiling (Singh et al., 2021). It is also clinically observed that in cases of MMR defects, the ATCs are likely to be associated with high mutational loads, the exact contribution of these mutations to thyroid cancer development is yet to be well understood (Tirro et al., 2019).

NGS has further enriched the insights of genomic changes in driving thyroid cancer, but a number of more advanced tumors, so-called dark matter, still do not have identified drivers (Cetti, 2017). Repetitive copy number alterations and preclinical models indicate dysregulated pathways, such as MAPK/ERK, PI3K/AKT/mTOR, and DNA repair, as primary factors to tumor progression and resistance to therapy (Bou Antoun and Chioni, 2023). The problem of limited availability of specific inhibitors and complexities of trials design still limits clinical translation. Genomic insights are potentially important in therapeutic development, as evidenced by recently approved targeted therapies against BRAFV600E and RET (rearranged during transfection). Further tumor profiling and model building is likely to provide insight into pathway crosstalk and inform strategies to bypass therapeutic resistance (Choi and Chang, 2023).

## 3 Tumor targeting mechanisms

The therapy of the thyroid cancer through tumor targeting relies upon the molecular and physiological processes of the cancerous cells of the thyroid gland to ensure both the efficiency and specificity of the diagnostic and curative treatments. These two are passive targeting and active targeting to improve the permeation and retention effect and the ligand-receptor interactions respectively as discussed below.

### 3.1 Passive targeting (enhanced permeation and retention effect)

The abnormal pathophysiological characteristics of tumor vasculature mainly dictate passive targeting of nanomedicines. The increase in tumor and angiogenesis rates leads to the disorganized endothelial structure, porous capillaries, incomplete basement membrane, and disrupted lymphatic drainage (Dudley and Griffioen, 2023). These characteristics all allow NPs to extravasate via vascular gaps and be concentrated in tumor tissues to a greater extent, a phenomenon called enhanced permeation and retention (EPR) effect. Physicochemical characteristics of nanocarriers, specifically particle size, surface charge, and hydrophobicity as well as stability, affect the efficiency of passive targeting to a great extent. Nanoparticles within the optimal size range (less than 10 nm) demonstrate improved tumor accumulation, as smaller particles are rapidly cleared by renal filtration, while larger particles are sequestered by macrophages or healthy organs (Chaurasiya et al., 2016). In addition, prolonged blood circulation time is critical for effective passive targeting, as it increases the likelihood of nanoparticle transport across tumor vasculature. Strategies such as steric stabilization, most notably PEGylation, have been employed to reduce rapid clearance by the reticuloendothelial system and enhance circulation longevity. However, emerging evidence of PEG immunogenicity and anti-PEG antibody formation highlights the need for alternative surface modification strategies to sustain passive targeting efficiency and therapeutic efficacy (Zhang et al., 2018). Generally, the EPR effect is weak in thyroid cancers because of the small size and well-differentiated vasculature. The anaplastic tumors show only modest vascular permeability which makes the passive accumulation inconsistent.

### 3.2 Active targeting (ligand-receptor interactions)

Active targeting enhances nanoparticle accumulation at tumor sites through specific ligand-receptor interactions on target cells. Under this method, nanocarriers are modified with ligands, antibodies or biomolecules that specifically bind receptors which are overexpressed on cancer cells, tumor-related endothelial cells or immune cells. The ligand binding induces receptor-mediated endocytosis that allows the uptake of nanoparticles into the cells and release of the drug in endosomal compartments. This approach enhances therapeutic accuracy by raising the concentration of the drug at target site with minimal off-target side effects (Large et al., 2019). A number of studies have shown much improved cellular uptake and tumor accumulation with active targeted nanoparticles in comparison to non-targeted systems. Nonetheless, active targeting must be effectively targeted to ensure that the nanocarriers fulfill the requirements of passive targeting such as an appropriate size, stability, and long circulation, and efficient drug retention. One of the key issues in active targeting is the development of protein corona after nanoparticle exposure to biological fluids that can change surface properties, conceal targeting ligands, and randomly affect biodistribution, cellular uptake, and toxicity (Anarjan, 2019). In addition to conventional cell targeting, transcytosis-based nanomedicine represents an advanced active targeting strategy, enabling nanoparticles to traverse biological barriers by exploiting cellular transport mechanisms. By integrating ligand-mediated recognition and barrier-crossing capabilities, active targeting strategies offer a refined approach to overcome tumor microenvironmental barriers and improve the delivery efficiency of the nanomedicines (Li A. et al., 2025). Many studies on thyroid cancer utilize the ligands to improve the uptake process, for example, the TSH or TSHR-binding peptides on nanoparticles, which bind the abundant TSHR (Xu et al., 2025). Similarly, the radioiodine based and the NIS-targeted agents exploit the sodium iodide symporter. As a result, the anti-VEGF antibodies on the nanomedicines enhance the uptake in vascularized anaplastic thyroid tumors (Zhang et al., 2019). Unlike the passive EPR targeting, which is nonspecific and gives low thyroid localization, the active targeting provides higher tumor-to-normal ratios but depends on the expression of the heterogeneous receptor (Xu et al., 2025). Although the active targeting can penetrate the cells *via* receptor-enhanced endocytosis, EPR alone may cause off-target uptake. However, the active ligands also have limitations for their complexity and potential immunogenicity.

## 4 Limitations of conventional monotherapy in thyroid cancer

Comprehensive molecular profiling of advanced thyroid carcinomas is used to identify actionable targets; however, clinical benefit is often limited by the emergence of acquired drug resistance and reduced progression-free survival. Resistance commonly arises through pathway compensation, whereby inhibition of the primary oncogenic driver is bypassed by activation of alternative signaling routes, most frequently converging on the mitogen-activated protein kinase (MAPK) pathway (Lee et al., 2020). Although selective RET (rearranged during transfection) kinase inhibitors represent a major advance due to a favorable safety profile and improved target specificity, resistance mechanisms driven by secondary mutations and bypass signaling have been increasingly reported (Shabbir et al., 2023). Similar evolutionary pressures have necessitated multiple generations of anaplastic lymphoma kinase (ALK) and neurotrophic tyrosine receptor kinase (NTRK) inhibitors to expand inhibitory coverage and counteract resistance. Given the dynamic molecular evolution of tumors under tyrosine kinase inhibitor (TKI) therapy, serial molecular profiling through tissue or liquid biopsy is essential to identify emerging resistance mechanisms (Yang et al., 2022). Since several resistance mechanisms can exist in one patient, proactive detection of driver changes and countermeasures signaling is essential to maximize the effectiveness of targeted therapeutic interventions and achieve long-lasting clinical outcomes (Garg et al., 2024).

The growing range of targeted therapy of thyroid cancer has enhanced the disease management and progression-free survival of various histological forms of the disease; nonetheless, systemic toxicity and off-target effects are still major clinical challenges. Although personalized medicine is now enabled by genomic markers and TERT promoter mutations, which have helped provide more selective treatment options, the existence of heterogeneous responses and adverse effects of treatment remain a limitation to the achievement of long-lasting benefit. Multikinase inhibitors, such as lenvatinib and cabozantinib, have shown better results in some of the subtypes, but are often linked with off-target inhibition of non-tumor kinases, which add to the toxicity and discontinuation of the treatment (Alqahtani, 2021). Despite the fact that newer agents and immunotherapies are more tolerable than traditional chemotherapy, the occurrence of resistance and immune-related adverse events highlights the necessity of using safer treatment methods (Bandara et al., 2025). The drug delivery systems that are based on nanoparticles provide an approach to minimize the systemic exposure and off-target toxicity through sustained tumor-selective release of the drug. The continuous clinical trials are also working to perfect these strategies to enhance outcomes in patients with aggressive and advanced forms of thyroid cancer. Locally advanced and metastatic anaplastic thyroid carcinoma (ATC) is still a form of malignancy that has been associated with poor prognosis in the past despite the urgent need to develop combinational and molecularly targeted therapeutic interventions (Abdalla et al., 2024). The accumulated clinical data over the recent years indicates that monotherapies are less effective in terms of survival rates, whereas the combination treatment, especially the two-targeted therapy and the combination of targeted kinase inhibitors with immunotherapy, shows a higher response rate and disease control. It is important to note that BRAF and MEK inhibition have demonstrated a strong efficacy in patients with actionable mutations indicating the oncogenic reliance of ATC on the mitogen-activated protein kinase (MAPK) pathway (Xiang et al., 2024). In a comparable fashion, a combination between angiogenesis inhibitors, e.g., lenvatinib, and immune checkpoint blockade (pembrolizumab) showed better objective response rates and overall survival than the historical data of tyrosine kinase inhibitor monotherapy (Song et al., 2020).

The common occurrence of the actionable mutations in ATC such as BRAF (V600E), RET, RAS, PIK3CA and TERT promoter mutations has led to widespread next-generation sequencing (NGS) based molecular profiling that has become the cornerstone of maximizing treatment selection. Nevertheless, the potential of precision-based combination therapies remains unexploited because of the underuse of genomic testing in clinical practice. Moreover, given the heterogeneity of resistance mechanisms and variability in the expression of immune biomarkers like programmed death-ligand 1 (PD-L1), it is important to recognize that combination approaches as opposed to single-agent agents are necessary to overcome pathway redundancy and immune escape (Tonella, 2025). Besides that, multimodal treatment paradigm has been supported by integration of systemic therapies with locoregional therapies like hypofractionated radiotherapy which has shown encouraging results. Although response rates have increased, overall survival is not very high, and response toxicity with different regimes is also different, which supports the significance of optimized regimens (Pestana and Ibrahim, 2020).

The conventional monotherapies for thyroid cancer often fail in the advanced cases due to diverse mechanisms of resistance. Some major factors causing resistance include the efflux transporters, poor penetration, an immunosuppressive microenvironment, cancer stem cells, and the genetic mutations (Zhang Y. et al., 2022; Yao et al., 2020). The nanoparticle carriers can overcome such delivery-related limitations by their enhanced permeability, retention, and targeting, which improve tumor uptake and can bypass the efflux pumps (Yao et al., 2020). For example, the lipid-peptide nanoparticles delivering the NIS mRNA restored the uptake of iodide in the anaplastic thyroid models (Li Q. et al., 2023). The nanocarriers have the ability to co-deliver combinations of multiple drugs to reverse the chemoresistance (Yao et al., 2020; Kumbhakar et al., 2025).

## 5 Nanomedicines as a therapeutic platform in thyroid cancer

Nanomedicines are nanoscale systems developed to integrate diagnostic and therapeutic functions within a single platform, commonly referred to as nanotheranostics. In thyroid cancer, these systems are designed to deliver therapeutic agents alongside diagnostic molecules, enabling simultaneous disease detection, monitoring, and treatment (Li L. et al., 2023). The properties of nanomedicines are governed by factors such as particle size, surface charge, porosity, density, stability, protein adsorption, and surface functionalization. Nanostructures are commonly functionalized with biocompatible polymers or targeting moieties, and can be programmed by external stimuli, such as pH change, temperature, ultrasound, or chemical signals, to provide site-directed and controlled drug delivery (Tang et al., 2018). Their biodistribution and bioavailability is based on the route of administration and physicochemical properties and enables them to have selective accumulation in tumor tissues. Nanomedicines also allow the incorporation of several targeting and imaging features in the same carrier, which promotes molecular imaging, tracking-assisted surgery, and personalized therapeutic intervention in the management of thyroid cancer (Gulwani et al., 2024).

Nanomedicines overcome some of the shortcomings that are linked with the treatment of thyroid cancer in comparison to traditional therapeutic methods; these are; low drug bioavailability, non-specific distribution, systemic toxicity, and resistance to treatment due to tumor heterogeneity and adaptations to the microenvironment (Hamidi et al., 2023). Nanocarriers shield therapeutic agents against untimely degradation, increase the water solubility of drugs with low solubility, and enhance penetration and diffusion into the tissues. Their long circulation time helps them to maintain constant and regulated release of drugs, minimizing drug adverse effects and maximizing therapeutic effects (Farah and Farah, 2019). Nanomedicines are also used to co-deliver diagnostic and therapeutic molecules, which can be used to monitor the distribution and treatment response of drugs in real-time. Nanomedicines address the drawback of the conventional treatment, which is the one-size-fits-all approach, by integrating imaging, targeted therapy, and customized treatment strategy within the same system, and enable the management of thyroid cancer in a precise manner (Dey et al., 2024).

### 5.1 Functionalization strategies of nanomedicines

Some of the functionalization methods of the nanomedicines include surface modification methods and the functional ligands applied in thyroid cancer targeting as discussed below.

#### 5.1.1 Surface modification techniques

Nanomedicines take advantage of physicochemical, optical, magnetic, as well as thermal characteristics of nanomaterials, thus an essential step in enhancing the diagnosis and treatment of malignancies, such as thyroid cancer. Traditionally, the clinically approved nanomedicines, including Doxil, were largely based on passive targeting through the EPR effect that enables selective concentration of NPs in tumor tissues, owing to leaky vasculature and compromised lymphatic drainage. Passive targeting has been proven by clinical studies to enhance intratumoral drug concentration and decrease systemic toxicity. Nevertheless, the heterogeneity of thyroid tumors (papillary, follicular, medullary, and anaplastic thyroid cancers) cannot be tackled by passively targeting with nanoparticle delivery efficiency depending on variability in vascularization, receptors expression, and tissue microenvironment (Wang et al., 2023). As a result, active targeting facilitated by surface engineering has become a paramount approach to enhance specificity of localization as well as therapeutic outcomes. Surface modification methods such as PEGylation to increase circulation time and steric stabilization and functionalization with ligands to specifically target tumor-specific biomarkers. Imaging-based nanomedicines, such as SPIO (Superparamagnetic Iron Oxide) nanoparticles for MRI, exemplify clinically relevant applications where surface modification enables non-invasive monitoring of nanoparticle biodistribution and treatment efficacy in thyroid cancer (Uppalapati et al., 2024).

The surface modification of the nanomedicines can be carried out by the covalent conjugation or non-covalent coating (Boehnke et al., 2020; Wang and Zhang, 2023). The covalent linking develops durable bonds and provides a stable attachment of the ligand between the surface of the nanoparticles and the functional groups. Examples of covalent conjugation include amide coupling, thiol-maleimide linking, silanization and covalent PEGylation. The EDC/NHS coupling creates amide bonds, and it is ubiquitous for attaching amines and carboxyl functional groups. Covalent PEGylation, e.g., by using the PEG-succinimidyl esters, creates a dense hydrophilic layer that greatly prolongs the circulation time and reduces opsonization (Cahn et al., 2025; Freire Haddad et al., 2022). The non-covalent approaches include attaching the surface moieties by weak interactions to enable simple and reversible functionalization. Examples of this technique include the electrostatic adsorption, hydrophobic insertion, the host-guest complexes, affinity binding, and the layer-by-layer assembly. This approach avoids complex chemistry and can preserve the activity of the ligand. For example, Boehnke *et al.* adsorbed the cationic tumor penetrating peptides onto the negatively charged layer-by-layer assembly of nanoparticles that retained the function of the peptide, however, non-covalent coatings are less stable *in vivo* than covalent conjugations (Boehnke et al., 2020).

#### 5.1.2 Functional ligands used in thyroid cancer targeting

Surface functionalization enables the nanoparticles to target molecular signatures that thyroid cancer cells have, which increases the specificity of the tumor and therapeutic efficacy. The functional ligands may be ranked into antibodies, peptides, and small molecules (Thiruppathi et al., 2017).

##### 5.1.2.1 Antibodies

Nanoparticles conjugated to monoclonal antibodies that target overexpressed receptors or tumor-associated antigens in thyroid cancer (e.g., RET, VEGFR, or HER2 in some aggressive subtypes of thyroid cancer) have been used to induce receptor-mediated endocytosis. Antibody-functionalized NPs increase tumor accumulation, enhance intracellular delivery of drugs and reduce off-target toxicity. Preclinical evidence shows that antibody-conjugated NPs have a much higher intratumoral concentration than non-targeted analogs despite limited clinical 3 translation due to immunogenicity and complicated production needs (M Cardoso et al., 2012).

##### 5.1.2.2 Peptides

Another small, chemically stable substitute of antibodies is the peptide based ligands. Peptides are able to selectively target tumor angiogenesis or proliferation, or tumor metastasis receptors in thyroid cancer. Peptide-functionalized nanoparticles retain specificity in targeting and reduce immunogenicity and steric hindrance, and therefore are the ideal multifunctional nanomedicines that incorporate both therapeutic and imaging capabilities (Omidian et al., 2025).

##### 5.1.2.3 Small molecules (folate, TSH-Based ligands)

Small-molecule ligands This category of ligand uses the over-expression of folate receptors or TSH receptors in thyroid tumors. The nanoparticles targeted with folate have been shown to exhibit improved cellular uptake and cytotoxicity in models of receptor-positive thyroid cancer whilst TSH-conjugated nanoparticles presently selectively deliver chemotherapeutics to thyroid tissue decreasing their systemic exposure. These ligands are of low immunogenicity, chemically stable, and can be produced by large-scale synthesis, which favors their use in the clinic (Davies and Latif, 2015).

To a great extent, the array of therapies of thyroid cancer is more specific and effective with the incorporation of targeting ligands on nanoparticle surfaces. We have ligand-mediated recognition that provides a preferential targeting of the therapeutic agents to malignant thyroid tissues and eliminates exposure to healthy organs and reduces systemic toxicity. It can also be used in the delivery of drugs to the intracellular level by receptor-mediated endocytosis, endosomal escape, and regulated drug release, which is especially crucial in aggressive thyroid cancers resistant to standard treatment (Banushi et al., 2023). Although functionalization enhances targeting of tumors, it also presents some difficulties such as changes in the pharmacokinetics, immunogenicity, and a possibility of masking of the ligands by protein corona. This is currently being addressed by ongoing studies on nanomedicines in the treatment of thyroid cancer to increase ligand density, nanoparticle physicochemical properties, and surface chemistry in order to surmount these obstacles and improve clinical translation of multifunctional, surface-modified nanomedicines to precision therapy (Abdelkawi et al., 2023).

## 6 Co-targeting multiple pathways using functionalized nanomedicines

New nanomedicines are under development that capitalise on the signaling pathways of thyroid cancer, e. g., the DNA based tetrahedral nanostructures of BRAF V600E-targeted siRNA silenced the MAPK/ERK pathway of the BRAF-mutant thyroid cancer by causing apoptosis (Zhang S. et al., 2025). Also, the polydopamine nanoparticles functionalized with the RGD peptides and doxorubicin facilitated the uptake of the tumor mediated by the receptor and targeted the integrin-rich thyroid tumors (Yuan et al., 2024). Multicellular nanomedicines have a strategic opportunity to co-target several cancer pathways in thyroid cancer, which addresses the heterogeneity of tumors and their resistance to therapy. These systems facilitate specific control of complementary molecular targets by designing nanocarriers to be able to deliver, at the same time, chemotherapeutic agents, genetic modulators, and RNA-based therapeutics (siRNA/miRNA). The synergistic cytotoxicity between chemotherapeutic and gene-/RNA-based payloads that is achieved through co-delivery improves apoptosis and compensatory survival signaling in tumor cells. As an example, tyrosine kinase-inhibitors and oncogenic transcript siRNA co-encapsulated into polymeric or lipid-based nanocarriers have been shown to increase tumor proliferation, angiogenesis, and metastasis inhibition in preclinical models of thyroid cancer (Chitkara et al., 2016; Zare et al., 2022). Additional assurance of spatially and temporally controlled liberation of the drug in the tumor microenvironment to reduce off-target toxicity is the incorporation of stimuli-responsive release mechanisms. Nanotechnology has revolutionized the biosensing and diagnostic methods of thyroid cancer by using NP unique physicochemical properties to improve sensitivity, specificity and multifunctionality. Nanomaterials such as metallic NPs, carbon-based nanomaterials, quantum dots, and carbon nanotubes have already been considered widely as diagnostic, imaging and therapeutic platforms (Hosseini et al., 2023).

### 6.1 Metal nanoparticles (AuNPs and AgNPs)

Gold nanoparticles (A У NPs) have a high surface-to-volume ratio, biocompatibility, and optical/electronic properties that can be controlled, which makes it useful in imaging, targeted therapy, and biosensing. These strategies include the iodinated Au nanoclusters functionalized with bovine serum albumin (BSA), which enable fluorescence/CT dual-modality imaging and accurate thyroid tumor detection in patient-derived xenograft models (Su et al., 2020). BSA-coated AuNPs and PFH-AuNPs conjugated with ligands (e.g., C225 antibody, TSHR ligands) selectively deliver drugs or gene therapies to thyroid cancer cells while enabling non-invasive imaging (P et al., 2025). PMAA-grafted AuNPs improve the efficacy of low-dose radioiodine therapy in tumor-bearing animal models, demonstrating synergy of nanocarriers with conventional treatments (Le Goas et al., 2019). Polydopamine-coated Au-Ag NPs accumulate in mitochondria of papillary thyroid carcinoma (TPC-1) cells, inducing mitochondrial dysfunction and apoptosis under low-intensity focused ultrasound (LIFUS) (Zhang D. et al., 2022). TSHR-targeted AuNPs can quantify TSH *via* localized surface plasmon resonance (LSPR), enabling rapid and sensitive hormone detection. Silver nanoparticles (AgNPs) also demonstrate anticancer potential by inducing reactive oxygen species-mediated apoptosis in thyroid cancer cells (Fayadh and Mohammed, 2022). Nevertheless, the metal nanoparticles are non-biodegradable and cause long-term toxicity by accumulating in liver, spleen, and the kidneys (Jakic et al., 2024; Ferdous and Nemmar, 2020). The AuNPs clear slowly and induce chronic inflammation and fibrosis in the organs (Jakic et al., 2024), whereas the AgNPs present dose-dependent organ toxicity (Ferdous and Nemmar, 2020).

### 6.2 Carbon nanomaterials

Carbon-based nanomaterials are clinically approved for lymph node mapping in papillary thyroid cancer, improving lymph node retrieval while preserving parathyroid function. Carbon nanoparticles convert near-infrared light into heat for localized tumor ablation, enhancing surgical outcomes and minimizing postoperative complications (Zhao et al., 2017). However, carbon-based nanomaterials, e.g., the graphene derivatives, have uncertain clearance and induce oxidative stress and inflammation (Ou et al., 2016).

### 6.3 Quantum dots (QDs)

Quantum dots have high-intensity fluorescence and tunable emission, which allows detection of thyroid cancer biomarkers with sensitivity. The QDs have been used to detect mutagenesis of the KRAS gene and used conjugated with JT-95 IgM antibodies to detect thyroid carcinoma specific protein. Sulfur-doped graphene carbon nitride QDs enhance signal processing for enzyme-free radiometric biosensors, improving sensitivity for genetic diagnostics (Li L. et al., 2023). But a major drawback of the standard QDs is that they usually contain heavy metals, are non-biodegradable, and also accumulate in liver or the kidneys where they cause dose-dependent oxidative and genotoxic damage (Cristian et al., 2024).

### 6.4 Carbon nanotubes (CNTs)

Selective binding to papillary thyroid cancer cell is made possible by functionalization with the TSHR antibodies or thyrotropin. Localized cell death (>60–70% *in vitro*) of tumor cells in multi-walled CNTs is achieved by laser activation, and recurrence is decreased in xenograft models. CNTS enable a high level of drug loading and light-to-heat conversion, although toxicity varies with length, diameter, and surface functionalization, and may have genotoxic, inflammatory, and pulmonary outcomes (Dotan et al., 2016). CNTs also possess fibrous and biopersistent character, and this is one of the significant drawbacks of their usage in the treatment of th thyroid cancer as they can trigger the prolonged inflammation, fibrosis and carcinogenicity in lungs (Kobayashi et al., 2017).

### 6.5 Polymeric and lipid carriers for thyroid cancer therapy

The recent developments in the treatment of thyroid cancer have been using polymeric and lipoid-based nanocarriers, which have been combined to achieve good efficacy with minimal systemic toxicity. Natural and synthetic polymers and lipid-based systems have all shown greater promise as multifunctional nanomedicine in thyroid malignancies (Silva et al., 2019).

#### 6.5.1 Liposomes

**FIGURE 3:**
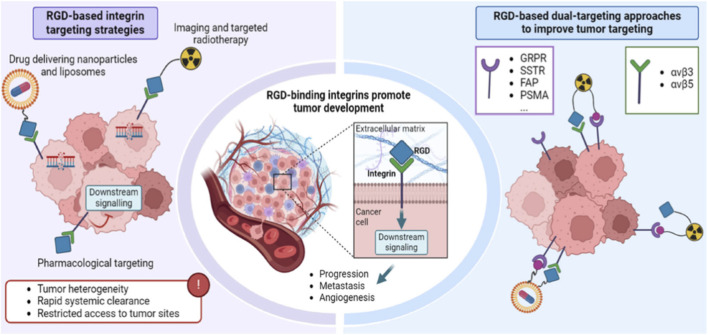
Demonstration of tumors with high-expression of RGD-binding integrin, which are the major regulators of tumor growth, angiogenesis, and metastatic dissemination. The schematic is a summary of integrin-targeted approaches, such as nanoparticle- and liposome-based drug delivery, radiopharmaceutical-guided imaging and radiotherapy, and application of RGD-based inhibitors. It further points out the dual-targeting strategies that can simultaneously target RGD-binding integrins and complementary molecular signaling, which increases diagnostic accuracy and therapeutic efficiency improvements compared to traditional single-target therapies (Bogdanović et al., 2024).

The phospholipid vesicles that can entrap hydrophilic and lipophilic drugs, known as liposomes, are still one of the most widely researched nanocarriers in the field of oncology. Selective uptake through TSH receptor (TSHR) overexpressed on thyrocytes is improved by functionalization of thyroid-targeting ligands, e.g., thyroid-stimulating hormone (TSH). TSH-conjugated liposomes with gemcitabine or cisplatin have been preclinically tested and demonstrated enhanced thyroid-targeting behavior and increase antitumor effect with minimal side effects. On the same note, tumor-suppressive effects of miR-34b cationic liposomes were observed in anaplastic thyroid carcinoma through VEGF-A regulation. PEGylation also enhances the circulation time, and lowers reticuloendothelial system (RES) eradication, thus facilitating effective delivery of siRNA to papillary thyroid cancer cells (Froehlich and Wahl, 2021). Liposomes have been also conjugated to metallic nanoparticles (LIPo-MNPs) to be used in theranostics, where they are used to deliver drugs and provide imaging at the same time (Musielak et al., 2021). Figure 3 helps to understand the importance of RGD-binding integrins in tumor progression and outlines integrin-targeted and dual-targeting approaches to enhancing the accuracy of diagnosis and the efficacy of treatment of cancer.

Liposomes have emerged with multifunctional characteristics such as Targeted, stimuli-responsive, and nucleic-acid-loaded for thyroid cancer therapy. The ligand-targeted liposomes, e.g., conjugated with TSH, anti-VEGF antibodies, peptides or aptamers, exploit the overexpressed thyroid receptors to increase the uptake of the tumors. For example, the TSH-conjugated liposomes as carriers for chemotherapeutics have shown 3–4 times higher thyroid accumulation and uptake in the TSHR + cells than the non-targeted formulations (Xu et al., 2025). The stimuli-responsive liposomes present an effective avenue in the treatment of thyroid cancer by remaining intact in circulation, but they release the loaded drugs at the tumor site *via* stimuli, e.g., pH, redox conditions, enzymes, or heat (Zhai et al., 2021; Fouladi et al., 2017). Furthermore, the cationic or ionizable liposomes can carry the siRNA/miRNA/mRNA to the thyroid cancer cells, and protect the RNA from nucleases, prolong its circulation, and facilitate its endosomal escape. For example, the PEGylated liposomes delivering miR-34b-5p to the anaplastic thyroid carcinoma suppressed VEGF-A and also reduced the size of the tumors in the mouse models (Maroof et al., 2018).

#### 6.5.2 Niosomes

Niosomes are non-ionic surfactant vesicles stabilized by cholesterol, providing enhanced stability, biocompatibility, and encapsulation versatility. Nanometer-scale niosomes can accommodate both hydrophilic and hydrophobic drugs, and in preclinical studies, drug-loaded niosomes demonstrated higher cytotoxicity than free drugs. Despite the promising results, their applicability in the thyroid cancer is still underexplored and there is a need for further investigation (Pingale et al., 2023).

#### 6.5.3 Polymeric nanotheranostics

Polymeric nanoparticles, including PLGA-based systems, offer multifunctional platforms for simultaneous diagnosis and therapy. Multifunction SHP2 or EGFR-functionalized PLGA nanoparticles were found to be very specific, accumulate drugs better and synergistically anticancer in thyroid cancer models (El-Hamm et al., 2022). The effectiveness of radioiodine treatment has been enhanced with hybrid polymer-metallic systems (e.g., PMMA-gold nanoparticles) and polyethylenimine-based polyplexes carrying EGFR-targeting peptides have been used to deliver sodium iodide symporter (NIS) genes to anaplastic thyroid carcinoma which have improved tumor control and survival in xenograft models (Schaffert and Ogris, 2013).

### 6.6 Multifunctional nanocarriers for localized thyroid therapy

#### 6.6.1 Nanoclay

**FIGURE 4:**
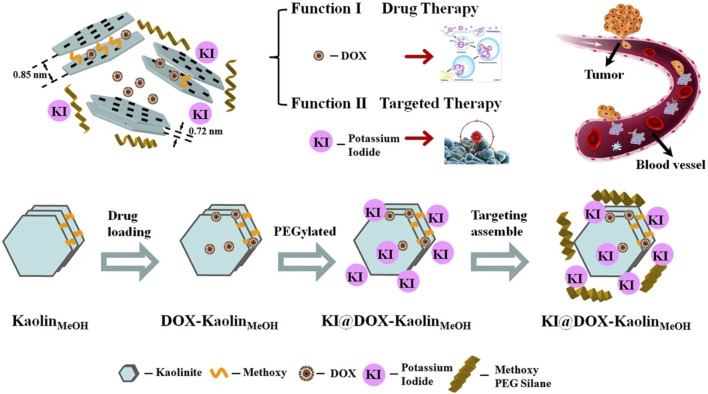
Representation of the sequential preparation of the KI@DOX-KaolinMeOH nanocomposite and the functional roles that it plays in tumor-based therapeutic strategies. Adapted from the reference (Zhang et al., 2016).

Phosphosilicate nanoclays ensure the retention of drugs and local concentrations, which are prolonged. Targeted Doxorubicin-loaded kaolinite nanoclay enhanced cytotoxicity in papillary and anaplastic thyroid cancer models (Liu et al., 2024). As an example, the stepwise synthesis of the KI@DOX-KaolinMeOH nanocomposite and its associated functional roles in tumor-targeted therapeutic applications is illustrated in Figure 4.

#### 6.6.2 Hydrogels

Hydrogels made of polymer can be used to release drugs on a localized basis and lasting. Hydrogel of Glycol chitosan and hyaluronic acid loaded with doxorubicin or quercetin has shown increased antitumor efficacy, synergy with kinase inhibitors and reduced exposure of the system to the host. Aplastic thyroid cancer treatment can be treated with drug release in hydrogel-micelle complexes controlled and released sequentially (Barbarisi et al., 2018).

#### 6.6.3 Wafers and nano-gels

Wafer-based biosensors and label-free SERS systems enable the diagnosis of thyroid tumors at an early stage with the help of miRNA or thyroglobulin biomarkers. Radio-sensitizers have mucoadhesive gels which enable the loco-regional therapy, maximizing the tumor deposition and reducing the systemic toxicity (Sahu et al., 2018).

#### 6.6.4 Patches for hormone delivery

Gel preparations of levothyroxine exhibit better absorption and bioavailability in hypothyroid models, which is evidenced by the absence of TT4, FT3, FT4, and TSH, and which deserve alternative options to oral therapy (Rancan, 2018). Polymeric and lipoid multifunctional nanocarriers have been shown to show a lot of potential in the treatment of thyroid cancer, incorporating targeted therapy, imaging, and localized drug delivery with little side effects in the entire system. They are versatile and can be co-delivered with chemotherapeutic, genetic, and RNA-based therapeutics, making them next-generation theranostic platforms (Froehlich and Wahl, 2021). Different nanoparticle systems along with their targets, mechanisms, and outcomes for therapeutic applications against thyroid cancer are critically analyzed in Table 1.

**TABLE 1:** A critical analysis of the different nanoparticle systems as well as their targets, mechanisms, and outcomes for therapeutic applications against thyroid cancer.

Nanomaterial	Target	Mechanism	Outcomes	References
Gold nanoparticles (AuNPs)	Papillary thyroid cancer cells (BCPAP, TPC-1)	Enhance drug delivery and imaging due to high surface area and biocompatibility	Promoted uptake and cytotoxic effects in thyroid cancer cell lines	Wang et al. (2019)
Mesoporous silica NPs	FTC-133 thyroid cancer cells	Surface modified with TSH ligand; controlled doxorubicin release under acidic conditions	Induced apoptosis *in vitro*; suppressed xenograft growth in mice	Li et al. (2017)
Kaolin nanoclay	Thyroid cancer cells	Encapsulates doxorubicin; controls diffusion	*In vitro* dose-dependent cytotoxicity; minimal off-target toxicity	Persano and Leporatti (2022)
Cisplatin-loaded lipid-polymer NPs	FTC-133 xenografts	TSH conjugation for tumor targeting; enhanced accumulation	4 times higher tumor accumulation; improved antitumor efficacy vs. non-targeted NPs	Liu et al. (2024)
Gemcitabine-loaded liposomes	TSHR-expressing CHO xenografts	TSH-targeted liposomes	3.5 times higher accumulation in xenografts; stronger antitumor activity than non-targeted liposomes	Paolino et al. (2014)
PAMAM-Dox@Lip-PIP-IR (PD@L-P-IR)	Tumor tissues	Core-shell nanoparticle; IR780 targets mitochondria; LIFU triggers controlled drug release; PIP inhibits MDR	Effective tumor targeting; enhanced drug release; synergistic anticancer effect	Zhu et al. (2023)
Liposomes encapsulating ATRA	Anaplastic thyroid cancer (ATC) cells (FRO)	PEGylated liposomes improve stability and bioavailability; encapsulates ATRA	Up to 80% reduction in FRO cell viability; limited effect on PTC-1 cells; selective differentiation therapy	Cristiano et al. (2017)
Traditional agents (RA, fatty acids, HDAC inhibitors, PPAR-γ)	Thyroid cancer cells	Stimulate NIS gene expression	Limited absorption, high dose required, lack of specificity	Shen and Chung (2005)

## 7 Preclinical landscape of Co-Targeted approaches for nanomedicines

The presence of a large amount of preclinical evidence in both *in vitro* and *in vivo* models supports the development of multifunctional nanomedicines to co target a variety of oncogenic pathways in thyroid cancer. Various nanoparticle systems have been considered in cell line-based studies on how they can be used to increase cytotoxicity and penetrate through drug resistance barriers in comparison to free drugs. As an illustration, it has been shown that liposomal delivery of traditional chemotherapeutic and genetic cargos like miR 34b 5p can have a significantly higher uptake and induce apoptosis in anaplastic and differentiated thyroid carcinoma cells and a significantly higher reduction in proliferation compared to non formulated agents. It has demonstrated the increased drug internalization and reduced efflux of doxorubicin targeted delivery *via* mesoporous silica or albumin stabilized nanoparticles leading to increased cytotoxicity of TPC 1 and other papillary thyroid cancer models *in vitro* and greater tumor suppression in assessed across standardized viability assays (Froehlich and Wahl, 2021).

These cellular effects have been translated into anti tumor outcomes with excellent safety profiles in animal models with systemic co delivery nanocarriers. In xenograft studies using thyroid cancer in immunodeficient mice, it has been found that nanoparticle mediated delivery of chemotherapeutic agents with common targeting moieties such as thyroid stimulating hormone (TSH) receptor ligand leads to enhanced accumulation of the tumor, delayed growth kinetics and decreased off target toxicity when compared to the same dose of free drugs. As an example, receptor directed co targeted delivery was proven by the 3 -4 fold uptake tumor and antitumor practicalities of TSH conjugated liposomal or lipid polymer hybrid nanoparticles in models of FTC 133 xenografts (Singh et al., 2025).

The co delivery systems tend to achieve a higher tumor volume reduction, survival period and reduce systemic adverse effects as compared to monotherapy controls in animal models. Certain formulations have also been demonstrated to modulate critical survival programs and drug resistance programs by raising intracellular drug concentrations, silencing drug efflux transporters or triggering pro apoptotic signaling. Despite the persistent difficulties in achieving a total eradication of tumors and the lack of translational studies, these preclinical results are considered to be substantial evidence that engineered nanocarriers with simultaneous multi pathway interactions can have a synergistic effect on suppressing the development of thyroid cancer with a better therapeutic index than traditional approaches (Li W. et al., 2023).

## 8 Translational challenges and clinical prospects

Converting multifunctional nanomedicines in the laboratory into clinical practice is a significant scientific/regulatory task with numerous overlapping issues. The first biological limitation is the inherent complexity of the tumor microenvironment and systemic physiological processes that may severely hamper the transport of nanoparticles, homogeneous tumor penetration and predictable biodistribution; dense extra-cellular matrices, malformed tumor vasculature and uneven expression of receptors across tumor regions can hamper the uniform administration of nanoparticles to tumors and can lead to heterogeneous clinical outcomes. Besides, biological mechanisms like protein corona and rapid immune recognition by macrophages decrease the circulatory time and targeting specificity, representing a safety issue, and lowering therapeutic index. Extensive toxicological analysis has noted the risk of organ retention, immune response, and oxidative stress, and detailed characterization of long-term biodegradation, clearance, and immunogenicity was considered to ensure patient safety (Maurya et al., 2025).

Language barriers are further complicated by technical and manufacturing barriers. The nanomedicine synthesis methods which work well on a small scale with consistency often fail to give consistent size, surface chemistry and drug loading when scaled to industrial scales, and variances in batch-to-batch can have a direct impact on pharmacokinetics, efficacy, and regulatory approvals. Good Manufacturing Practice (GMP) Standardization of production, powerful quality control procedures of thorough nanoparticle characterization, and scalable processes maintaining functional integrity are all necessary but not fully developed. In addition, expensive infrastructure and large-scale production of the nanomedicines increase the economic and logistical challenges such as high-cost raw materials, complex formulation design, and high storage requirements which have the potential to affect the stability and shelf life (Rodriguez et al., 2022). The large-scale processing parameters, e.g., the temperature, mixing, and the removal of the solvent, *etc.*, need to be highly controlled. Sourcing the raw materials, such as high-purity lipids, polymers, and biological ligands, is also another challenge. The high-pressure homogenization can improve uniformity, but it requires robust control on the process (Buya et al., 2024; Paliwal et al., 2014).

Regulatory wise, the lack of harmonized international standards of evaluation of nanomedicines significantly delays the clinical manifestation. Nanoparticles contrasted with conventional small molecules have size-dependent behaviors and multifunctionality that requires special consideration of safety, efficacy, and quality. The regulatory authorities, including the U.S. Food and Drug Administration (FDA) and European Medicines Agency (EMA) frequently demand a substantial amount of preclinical and clinical data, but the standardized methods of characterizing nanoparticles, their biodistribution measures, and long-term toxicity have not been developed, and this uncertainty in the approval process and delay the time between development and clinical access. Nanomedicines heterogeneity also makes it difficult to evaluate regulatory aspects, since it requires specific approaches to match physicochemical properties with biological performance (Mangla et al., 2025). The regulatory bodies expect the demonstration of control over the properties of the nanomedicines and the parameters of the process, with immunogenicity assessment (Mohamed et al., 2019), and bridging the research gap in the process variability. It is a fact that no thyroid-specific regulatory pathway exists and so the processing of the nanomedicines has to follow the standard rules.

Clinical trials are currently going on to optimize the dosing, biodistribution, and safety profiles, and to incorporate new design characteristics to overcome the barrier of biology and enhance specificity of targets. There is a growing acceptance of the importance of strategic collaboration between researchers, industry, regulatory organizations and clinicians to overcome translational barriers, and new efforts are aimed at developing uniform guidelines, enhancing predictive preclinical models and modeling the design of nanomedicines to clinical requirements. With the maturation of these endeavors, clinical opportunities in the field of nanomedicines in thyroid cancer and other solid tumors will continue to grow, and there will be potential to achieve precision, efficacy, and safety that will outshine the traditional therapeutic approaches (Alshehri et al., 2020). The thyroid cancer specific nanomedicines are yet to be approved by FDA, and the majority of proofs are repurposing the approved formulations or combination plans. However, there exist some instances of the nanomedicines that are undergoing clinical trials in thyroid cancer. PEGylated liposomal doxorubicin (PLD) in combination with cisplatin is one such example, in a Phase II trial in thyroid carcinoma. The other Phase II is the nab-paclitaxel, which is an albumin-bound paclitaxel, and is used in combination with the atezolizumab against the poorly differentiated thyroid cancers (Laetitia et al., 2020).

## 9 Future perspectives

Nanomedicines in thyroid cancer are becoming directed towards the future by customized therapeutic approaches that direct therapy to specific genomic, proteomic, and immunophenotypic signatures. More recent investigations have pointed at the opportunity of designing nanocarriers that incorporate patient-specific biomarkers into their targeting systems, allowing accumulation to be selective in tumors with specific molecular phenotype, including BRAF^V600E, RET, RAS or NIS expression phenotype. Combination of high-throughput sequencing data with nanocarrier design protocols has enabled the creation of custom nanomedicines that can react to the specific tumor microenvironmental signals, e.g., pH, redox gradients, and enzymatic activities. These targeted approaches to nanomedicines are likely to enhance treatment efficacy with reduced systemic toxicity especially in the heterogeneous subtypes of thyroid cancer where responsiveness to traditional therapy has broad variability. Recent preclinical results indicate that co-delivery approaches based on mutation signature show a markedly improved response compared to non-targeted preparations in orthotopic xenograft models of thyroid carcinoma (Wang and Zhang, 2023).

This type of synergy between nanomedicines and immunotherapy has demonstrated potential promise in preclinical models with nanocarrier-mediated combination therapy resulting in better infiltration of cytotoxic T lymphocytes, lower regulatory T cell populations, and better tumor regression in thyroid and other solid tumor models. In addition, molecular profiling-based paradigms of precision medicine are entering nanocarrier selection criteria and dosing schedules, maximizing therapeutic windows and reducing adverse events in individual patients. These integrative techniques are a development of blanket systemic therapy to biologically reasonable, mechanism-based treatment planning (Morchón-Araujo et al., 2025).

New applications and functionalization strategies of nanomedicines are still increasing with the identification of new targets and emerging applications. The most recent studies have focused on the functionalization of nanoparticles with ligands targeting tumor specific receptors that are not classified as traditional markers such as integrin subtypes, chemokine receptors, and metabolic transporters that are over expressed in aggressive thyroid cancers. Indicatively, peptide functionalization with tumor-associated fibroblast receptors or metabolic receptors was effective to improve tumor penetration and uptake in orthotopic thyroid models. Nanocarriers have been designed to respond to localized microenvironmental conditions by stimuli-responsive surface chemistries, including redox-reactive linkers and matrix metalloproteinase (MMP)-activated coats. This enables the deployment of drugs with high spatial accuracy, which lowers off-target toxicity and enhances pharmacodynamics (Ren et al., 2025).

Recent scientific consensus comprises of the emergence of universal *in vitro* and *in vivo* platforms able to forecast human pharmacokinetics and toxicity more faithfully, standardized methods of nanoparticle characterization (e.g., size, surface chemistry, and payload stability), and comprehensive reporting on the biological interactions (e.g., formation of protein corona and immune responses). The iterative design-testing cycles are also supported by researchers, which can combine the computational modeling process with the empirical validation to optimize the process fast without reducing the safety. Parallel to this, future nanomedicine clinical success requires collaborative clinical consortia with a mechanism-oriented trial approach of nanomedicines to translate promise to patient outcome. These attempts must focus on biomarkers of response and resistance, dynamic dosing, and real-time imaging surrogates to measure intratumoral distribution and therapeutic interaction (Fahim et al., 2025).

## 10 Conclusion

The current review indicates the changing perspective of nanomedicines in terms of their application in addressing biological complexity and therapeutic resistance of thyroid cancer. Recent progress in the engineering of nanoparticles, surface modification and pathway-based targeting has facilitated more specific diagnostic and therapeutic approaches than traditional systemic approaches. All these findings are a reiteration of the potential of nanomedicines to fill existing gaps in the current knowledge of molecules and effective clinical intervention in advanced thyroid malignancies. Co-targeted functionalized nanomedicines are important because they can overcome tumor heterogeneity and adaptive signaling networks that can inhibit the efficacy of single-agent therapies. These systems can provide a logical solution to compensatory signaling suppression or therapeutic resistance prevention by allowing co-delivery of chemotherapeutics, gene-based therapeutics or immune-modulating molecules in a single nanoscale platform. Targeting can be further increased by functionalization with thyroid-specific ligands, which decrease off-target toxicity, but do not decrease therapeutic potency. This combined approach is a transition to the standardized treatment paradigm to the paradigm of biologically informed, mechanism-based interventions in the management of thyroid cancer. Further interdisciplinary studies, which are backed by strong preclinical validation and well-planned clinical trials, will be essential in fully utilizing the therapeutic potential of the nanomedicines and in transforming them into long-term, patient-focused solutions to refractory thyroid cancers.
